# Climate response to the 8.2 ka event in coastal California

**DOI:** 10.1038/s41598-017-04215-5

**Published:** 2017-06-20

**Authors:** Jessica L. Oster, Warren D. Sharp, Aaron K. Covey, Jansen Gibson, Bruce Rogers, Hari Mix

**Affiliations:** 1 Department of Earth and Environmental Sciences, Vanderbilt University, 2301 Vanderbilt Place, PMB 351805, Nashville, TN 37235 USA; 2 Berkeley Geochronology Center, 2455 Ridge Road, Berkeley, CA 94709 USA; 3 Western Cave Conservancy, Santa Cruz, California USA; 4 Department of Environmental Studies and Sciences, Santa Clara University, Santa Clara, CA 95053 USA

## Abstract

A fast-growing stalagmite from the central California coast provides a high-resolution record of climatic changes synchronous with global perturbations resulting from the catastrophic drainage of proglacial Lake Agassiz at ca. 8.2 ka. High frequency, large amplitude variations in carbon isotopes during the 8.2 ka event, coupled with pulsed increases in phosphorus concentrations, indicate more frequent or intense winter storms on the California coast. Decreased magnesium-calcium ratios point toward a sustained increase in effective moisture during the event, however the magnitude of change in Mg/Ca suggests this event was not as pronounced on the western North American coast as anomalies seen in the high northern latitudes and monsoon-influenced areas. Nevertheless, shifts in the White Moon Cave record that are synchronous within age uncertainties with cooling of Greenland, and changes in global monsoon systems, suggest rapid changes in atmospheric circulation occurred in response to freshwater input and associated cooling in the North Atlantic region. Our record is consistent with intensification of the Pacific winter storm track in response to North Atlantic freshwater forcing, a mechanism suggested by simulations of the last deglaciation, and indicates this intensification led to increases in precipitation and infiltration along the California coast during the Holocene.

## Introduction

Greenland ice cores document an abrupt cooling event ~8200 years ago^1^. The “8.2 ka event” lasted ~160 years, is the most distinctive isotope excursion in the Holocene ice core record^2^, and is thought to be the result of suppressed Atlantic Meridional Overturning Circulation (AMOC) due to draining of glacial lakes Agassiz and Ojibway into the North Atlantic^3, 4^ or reorganization of North Atlantic Ocean and atmospheric circulation following collapse of the Laurentide Ice Sheet^5^. Records of the 8.2 ka event at lower latitudes help to delineate the response of near-modern climate to this perturbation. Although documenting the spatial extent and duration of the 8.2 ka event from proxy records outside of Greenland has been challenging due to the brevity of the event^6^, mounting evidence from mid-latitude and tropical records suggests cooling in the North Atlantic region^7^, and a southward shift of the Intertropical Convergence Zone (ITCZ) and associated precipitation bands^8^. In British Columbia, lake sediments suggest glacial advance, consistent with a cooler and/or wetter climate^9^, and marine sediments indicate decreased sea surface temperatures along the northern California coast^10^. At mid-latitudes in western North America, however, the 8.2 ka event has remained poorly characterized given a lack of records of appropriate temporal resolution. This is unfortunate, as the region’s response to a freshening of the North Atlantic under interglacial conditions is relevant to modeling possible future climate change in this hydroclimatically sensitive region.

**Figure 1:**
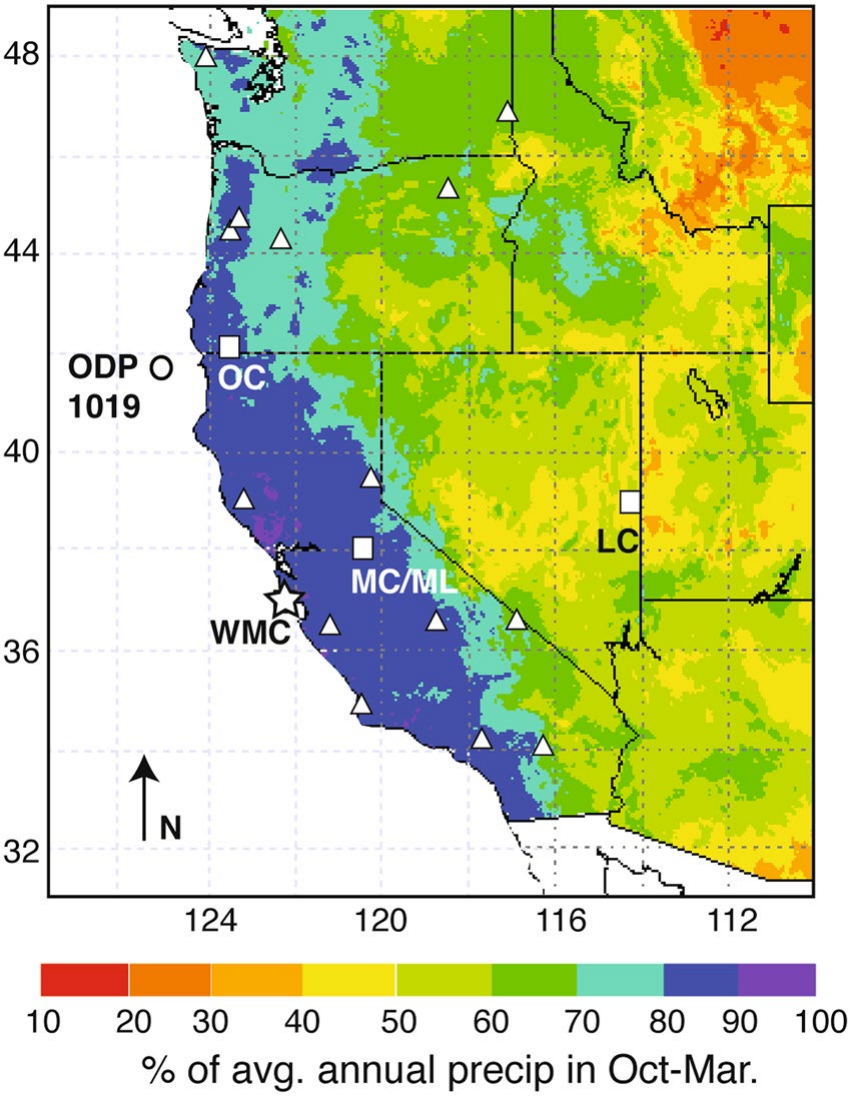
Location of White Moon Cave (star) and seasonality of modern precipitation in surrounding region. Boxes show locations of other Holocene speleothem records from the western United States: OC = Oregon Caves^20^, MC/ML = Moaning and McLean’s Caves^24^; LC = Lehman Caves^28^. Circle shows location of ODP core 1019^10^. Triangles show locations where event-scale δ^18^O_p_ has been analyzed^26^. Background map is the percent of average annual precipitation (1981–2010) that occurs during the cool season (Oct.–Mar) from the PRISM dataset^54^. Map modified from the Western Regional Climate Center^55^.

Here we present a new multi-proxy record from a fast-growing speleothem (WMC1) from White Moon Cave on the central California coast that precipitated prior to, during, and after the 8.2 ka event (Fig. 1). This record provides some of the first high-temporal-resolution evidence of the response of coastal California climate to the most distinctive climatic event of the Holocene. As shown below, the new record suggests that the 8.2 ka event was associated with a brief period of wetter conditions, potentially arising from increased storminess, and demonstrates a near synchronous climatic response to this event on both sides of the Pacific.

### Site and Sample Background

White Moon Cave (WMC) formed within late Paleozoic marble in the Santa Cruz Mountains near Davenport, CA (N37°00’, W122°11’, Fig. 1), approximately 18 km northwest of Santa Cruz. The cave entrance is located in the wall of an abandoned quarry that transects the natural cave, ~170 m above sea level. WMC1 (Fig. S2) is a 25.5 cm tall stalagmite collected >250 m from the modern entrance in the quarry wall and >350 m from the nearest natural entrance (Fig. S2). Petrographic analysis reveals that WMC1 consists of calcite displaying elongated columnar fabrics intercalated with fine layers of silicate detritus (Fig. S3)^11, 12^. Elongated columnar fabrics have been related to high seepage water discharge and commonly occur in speleothem from caves developed within rocks that contain dolomite or other Mg-rich phases^11, 12^.

The cave site experiences a warm-summer Mediterranean climate. The area receives on average 800 mm of rain annually, with >80% of this rain occurring in the cool season (Oct.–Mar.) (Fig. 1). Given its coastal location, the amplitude of seasonal temperature variability is small, with average winter temperatures of 11.3 °C and average summer temperatures of 18 °C^13^. Cool season rain comes in the form of winter storms, which may originate from the northern or mid-latitude Pacific. However, occasionally, this region is influenced by extra-tropical cyclones that draw moisture from the central or eastern tropical Pacific. These systems can develop narrow filaments of concentrated near-surface water vapor called atmospheric rivers, which are often associated with intense flooding along the Pacific coast^14^.

## Results

The stalagmite was cut into quarters along the growth axis (Fig. S2), eleven subsamples were dated using established U-Th techniques, and the stalagmite was analyzed for δ^18^O, δ^13^C, and trace elements including Mg, Sr, Ba, P, Y, Zn, and U (see Methods). Elemental concentrations are reported as ratios to calcium (mmolX/molCa). Results indicate that stalagmite WMC1 grew between ~8.6 and 0.24 ka (see Table S1). We focus herein on an interval of relatively rapid stalagmite growth (on average ~100 μm/year) around the 8.2 ka event, from ~8.6 to 6.9 ka. Ten samples from this interval were dated. Three were unsuitable for precise (or accurate) U-Th dating because of high levels of ^232^Th (probably derived from alumino-silicate detritus present in mm-scale voids). Seven other dates on samples of relatively pure carbonate have a median uncertainty of ±37 years and indicate stalagmite growth at a relatively constant rate from 8604 ± 34 to 6937 ± 32 cal yr BP (i.e., age before 1950; see Methods for further details, all errors 2σ). These dates were used to construct an age model for the proxy data via StalAge^15^ (see Fig. S4). The mean rate of extension along the growth axis of ~100 μm/year facilitated constructing proxy records of sub-annual (laser ablation) to multi-annual resolution (micromilling).

**Figure 2:**
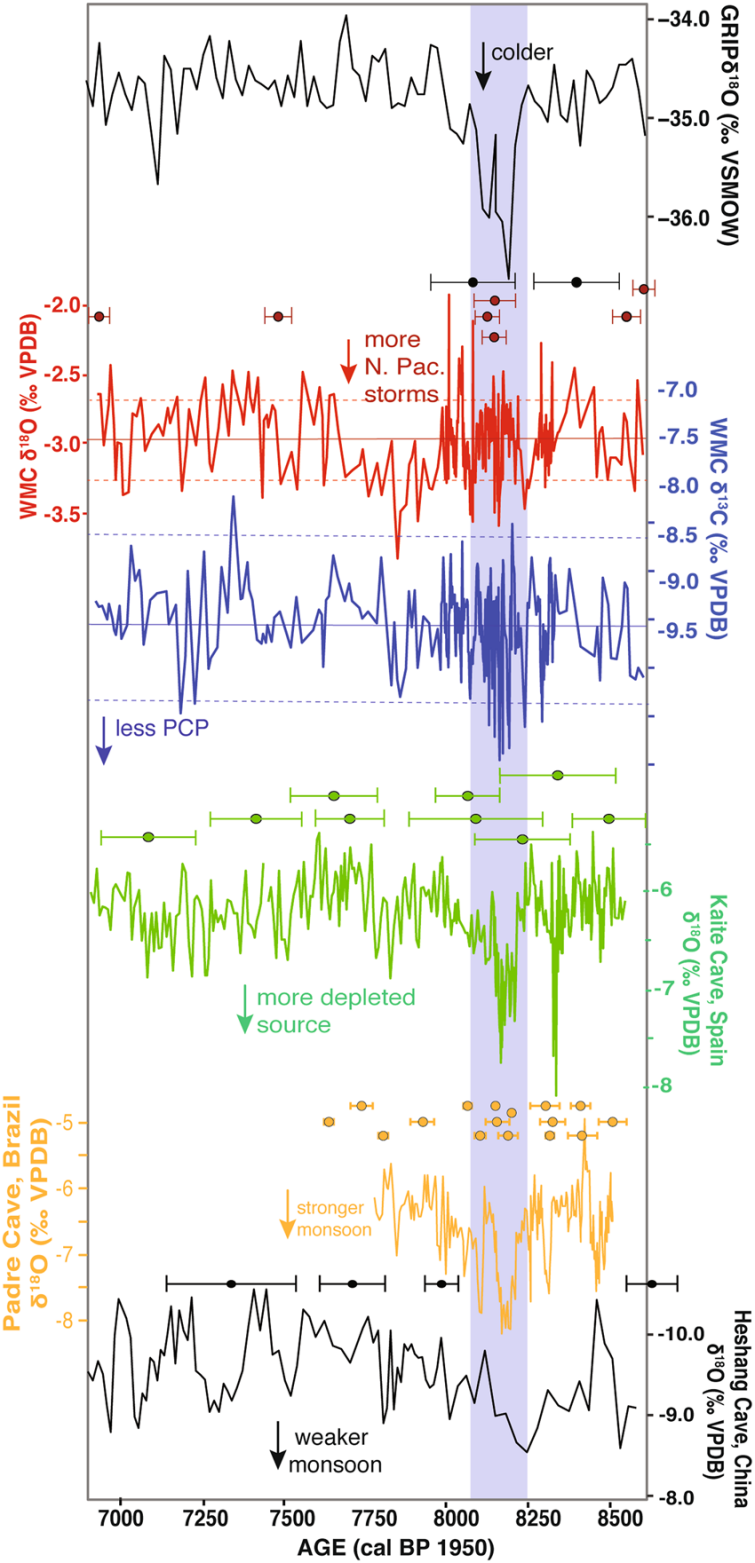
Select global proxy records of the 8.2 ka event compared to the WMC record. From top to bottom: GRIP ice core δ^18^O^56^; speleothem, δ^18^O (red), and δ^13^C (blue) from WMC (this study); and speleothem δ^18^O from Kaite Cave, Spain^41^, Padre Cave, Brazil^8^, and Heshang Cave, China^57^. Associated U-series ages and 2σ errors shown by circles and error bars above each speleothem record. For WMC, ages shown in black required large, model-dependent corrections for initial Th and are not used in the age model; date for highly detritus-rich sample AC-2 is not shown. The timing and duration of the 8.2 ka event based on the GRIP record is shown by the blue shaded bar. Dashed horizontal black lines delineate WMC records from this study. Solid and dashed red and blue lines for the WMC stable isotope proxies designate the mean values for the entire record and the 1SD (oxygen) and 2SD (carbon) ranges, respectively.

We acquired measurements of carbonate *δ* ^18^O and *δ* ^13^C from ~6900 to 8600 cal yr BP at sub-decadal to decadal temporal resolution and at higher, annual to bi-annual resolution in the vicinity of the 8.2 ka event, from ~8060 to 8340 cal yr BP. *δ* ^18^O varied between −1.92 and −3.83‰, and *δ* ^13^C varied between −6.73 and −9.45‰. The *δ* ^13^C record from WMC1 displays large amplitude, rapid variations during the 8.2 ka event **(**Fig. 2). The extremes of these excursions fall more than two standard deviations (SD) outside of the mean of the entire dataset (Fig. 2). Rapid shifts to the lowest *δ* ^13^C values observed in WMC1 occur near the middle of the 8.2 ka event, and these are synchronous within dating uncertainties with the central anomalies displayed in speleothem records of the event from Spain, Brazil and China (Fig. 2). The *δ* ^18^O record shows less overall variability, with slightly above-average values through the 8.2 ka event except for three shifts to lower values that last ~20–30 years each and occur at the beginning, middle, and just after the 8.2 ka event. These negative shifts fall outside 1 SD of the mean of the *δ* ^18^O dataset (Fig. 2). However, overall more negative *δ* ^18^O values occur after the 8.2 ka event between ~7650 and 7980 cal yr BP.

**Figure 3:**
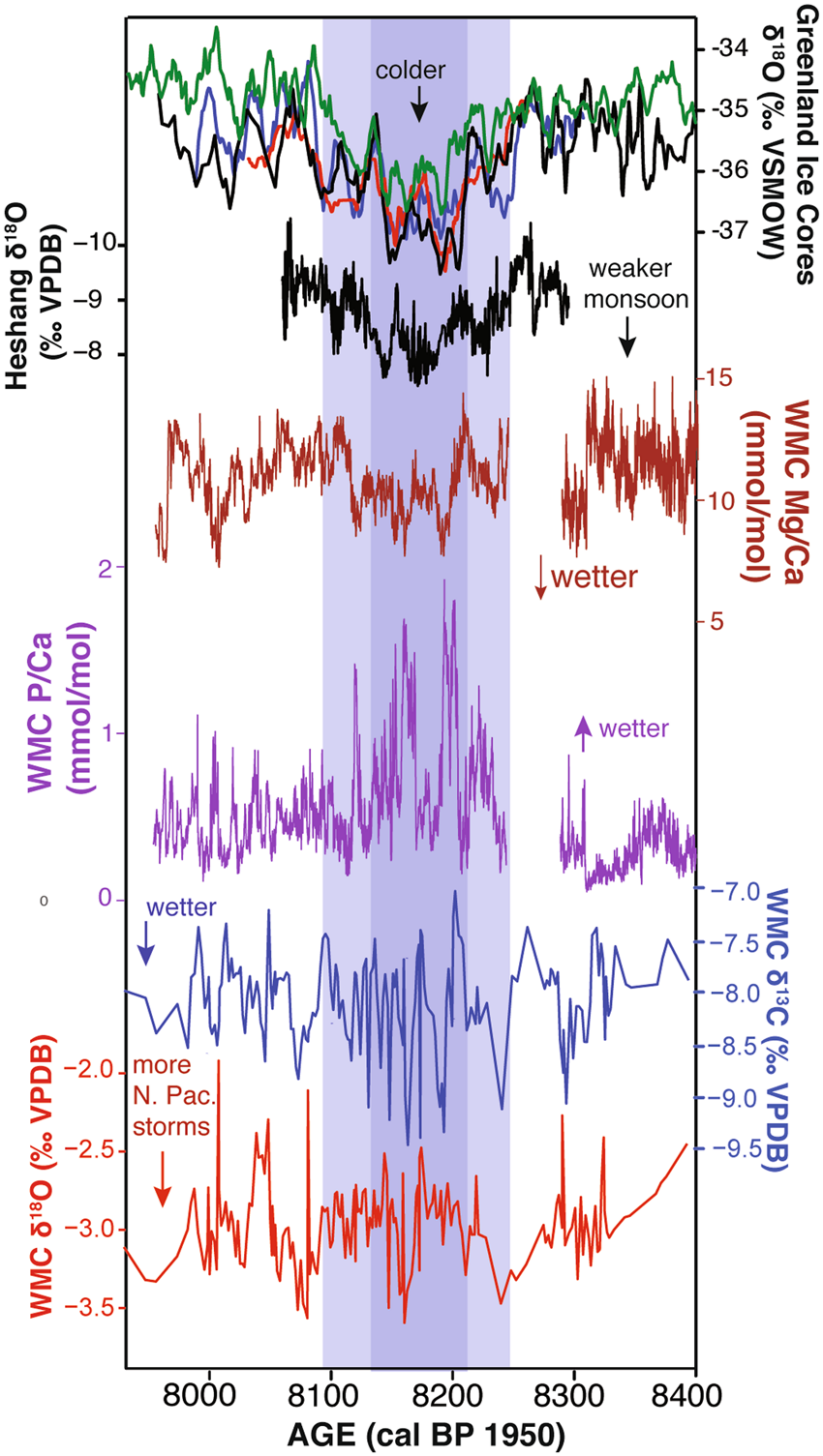
High resolution records of the 8.2 ka event. From top to bottom: Greenland ice core δ^18^O from GRIP (red), GISP2 (black), NGRIP (blue), and Dye 3 (green) on the GICC05 time scale^2^; the updated Heshang Cave δ^18^O record (black)^42^; WMC1 Mg/Ca (brown); P/Ca (purple); δ^13^C (blue); and δ^18^O (red) (this study). Dark and light blue shading depict, respectively, the central portion and entire duration of the 8.2 ka event as described in ref. 2 based on the Greenland ice core records.

We also measured trace element concentrations (Mg, Sr, Ba, P, Zn, Y, U) at sub-annual to annual resolution between ~7850–8650 cal yr BP. WMC1 displays a shift to sustained lower Mg/Ca and correlative, high amplitude oscillations in P/Ca across this interval (Fig. 3). Stalagmite Mg/Ca shows significant moderate negative Pearson correlations with Sr/Ca and Ba/Ca (*r* = −0.41, p < 0.001; *r* = −0.29, p < 0.001, from a two-tailed t-test, respectively). As discussed below, we interpret these relations in terms of moisture-controlled variations in soil and host rock inputs to epikarst solutions during the 8.2 ka event.

### Stalagmite Proxy Interpretations

WMC is a remote cave that was mostly dry during the course of this study due to the recent California drought, and thus it was challenging to obtain regular drip water measurements. However, the average *δ* ^18^O of drip water sampled three times at two locations within WMC in March and December 2015 and March 2016, is −5.2‰ (VSMOW) (1 SD = 0.4; n = 7) (Table S2). This falls within the range of measured *δ* ^18^O_*p*_ of monthly average rainfall in Santa Cruz collected between December 2015 and March 2016, of −3.03 to −5.37‰ (VSMOW) (Table S3). This value is also within the range of event-scale *δ* ^18^O_*p*_ values reported for Pinnacles National Park, ~145 km southeast of WMC, between 2001 and 2005 (−2.3 to −14.7‰, average of −6.8‰)^16^. Drip water *δ* ^18^O values also showed minimal variations between drip sites on a given day (−0.22 to −0.44‰) and showed the same direction of change at each site between each sampling interval.

Speleothem δ^18^O may be influenced by non-equilibrium isotope fractionation that can occur due to rapid degassing and calcite precipitation^17^. There is only a moderate positive correlation between *δ* ^18^O and *δ* ^13^C down the growth axis of WMC1 (*r* = 0.37, p < 0.001), lending evidence that these two proxies are not predominantly controlled by kinetic effects during calcite precipitation^18^. Using the fractionation relationship of ref. 19 that is calibrated for cave environments, and the average annual temperature in Santa Cruz (14.8 °C), we calculate a *δ* ^18^O of −4.3‰ (VPDB) for calcite precipitated in equilibrium with average modern drip water, and a range of −2.1 to −4.9‰ (VPDB) for calcite precipitated in equilibrium with the range of measured monthly average rainfall *δ* ^18^O_*p*_. Although present slow growth rates prohibit analysis of modern calcite, this range is similar to that of measured WMC1 *δ* ^18^O for the Holocene (−1.92 to −3.83‰ VPDB), and suggests WMC1 faithfully records drip water isotope values along its growth axis. The overall low range of variability in the WMC1 *δ* ^18^O record (~1.6‰) is also consistent with other Holocene speleothem records from the U.S. west coast^20, 21^. Given these findings, we believe that the *δ* ^18^O of WMC1 reflects local rainwater *δ* ^18^O (*δ* ^18^O_*p*_).

Although speleothem studies tend to rely heavily on records of *δ* ^18^O variability, the subdued nature of variations in WMC1 during the 8.2 ka event likely reflects regional complexities in the controls on *δ* ^18^O_*p*_ that have only recently been appreciated. Previous speleothem and cave drip water studies from Oregon^20, 22^ and central California^23, 24^ (Fig. 1) attributed water and speleothem δ^18^O variability to changes in local atmospheric temperature at the time of precipitation and moisture sources. Observations of modern event-scale δ^18^O_*p*_ at sites along the US west coast, including central California^16, 22, 25^ (Fig. 1) suggest that seasonal and interannual variability in *δ* ^18^O_*p*_ is due to varying proportions of moisture from subtropical (higher *δ* ^18^O_*p*_) versus mid-latitude and north Pacific-derived (lower *δ* ^18^O_*p*_) sources. In contrast, recent isotope-enabled modeling of seasonal and interannual variability in δ^18^O_*p*_ suggests that, although temperature and moisture sources are important elsewhere in the western US, variations in droplet condensation height are the dominant control on δ^18^O_*p*_ throughout most of California^26, 27^.

We analyzed monthly averaged rainwater samples from Santa Cruz for the winter of 2015–2016 and likewise found no significant relationship between *δ* ^18^O_*p*_ and temperature or precipitation amount (Table S3). With this small sample size, we were not able to discern the influence of moisture source on Santa Cruz *δ* ^18^O_*p*_, as March 2016, which experienced two large subtropical atmospheric river events, had the lowest value for *δ* ^18^O_*p*_ (Fig. S5). Thus, it is possible that the isotopic source signal at this site is overprinted by vapor condensation processes, as is suggested by isotope enabled models^26, 27^. The shifts to lower speleothem *δ* ^18^O during the 8.2 ka event, therefore, might reflect periods when more northerly-sourced moisture reached the cave site. However, the effects of source changes on *δ* ^18^O_*p*_ may be complicated by changes in droplet condensation height. For example, increased condensation heights, which would drive decreased *δ* ^18^O_*p*_ values, may occur in response to a more intense storm track that drives upper level divergence^27^. Thus, due to the complexity of the controls on *δ* ^18^O_*p*_, we posit that WMC1 δ^13^C values and trace element concentrations provide a more informative assessment of local climate response to the 8.2 ka event at this site.

Cave monitoring studies and speleothem records from similar semi-arid, mountainous regions in western North America including coastal Oregon^20^, the Sierra Nevada foothills^23^, and the Great Basin^28^ suggest that carbon isotope signatures in cave drip waters and speleothems reflect changes in water supply via their influence on soil processes and degassing in the epikarst and within the cave itself. The δ^13^C_DIC_ (DIC = dissolved inorganic carbon) of modern cave drip water was analyzed at three sites in WMC in December 2015 and March 2016. Values ranged from −2.73 to −7.23‰ (VPDB), and were 1 to 4‰ lower in March than in December with decreases occurring at all sample sites (Table S2). This direction of change is consistent with drip water δ^13^C_DIC_ in the Sierra Nevada which displays decreasing values from early winter through early summer when water supply is adequate to high and soil respiration is increasing^23^. The shifts in early Holocene WMC1 δ^13^C are likely too rapid to have arisen through changing proportions of C_3_ and C_4_ plants above the cave or through long-term changes in atmospheric *p*CO_2_^29^. In addition, with a mean annual precipitation of 800 mm, WMC presently falls outside the range where soil respiration rate is likely to be sensitive to changes in moisture^29^. Thus, we interpret the WMC1 δ^13^C record to reflect changes in water supply leading to variable degassing of CO_2_ and prior calcite precipitation (PCP) in the epikarst and cave, where preferential degassing of ^12^CO_2_ leads to higher residual δ^13^C_DIC_ values. We suggest that the 8.2 ka event at WMC was characterized by highly variable water supply, punctuated by large increases in infiltration that lead to sharply decreasing speleothem δ^13^C values in the core of the 8.2 event.

**Figure 4:**
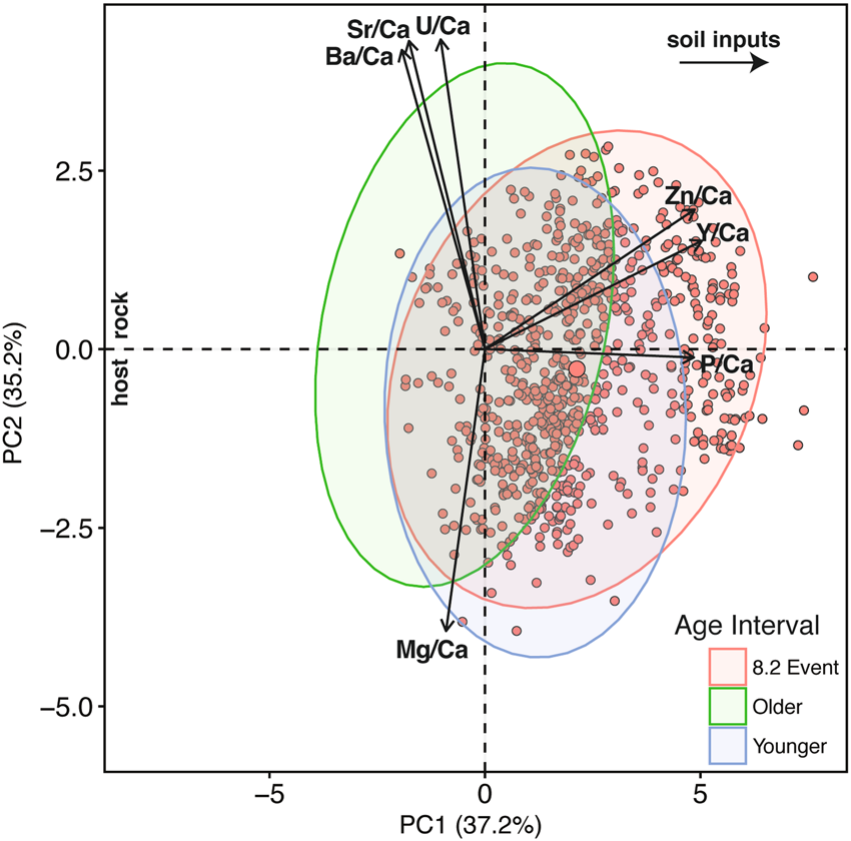
Principle components analysis of trace element variations in WMC1 showing PC1 and PC2. Results were log-transformed prior to analysis to account for non-normal distributions. 95% confidence ellipses are shown for the points that are older, younger, and coeval with the 8.2 ka event (~8250–8100 cal yr. BP). For simplicity, only points coeval with the 8.2 ka event are shown.

Trace element time series support the interpretation that climatic conditions were highly variable during the 8.2 ka event (Fig. 3). A principal components analysis (PCA) of the trace element concentrations in WMC1 reveals a first component (PC1) controlled by variations in the ratios of primarily soil-derived elements P, Zn, and Y to Ca, while a second component (PC2) is controlled by variations in the ratios of primarily host-rock derived elements Mg, Sr, and Ba to Ca (Fig. 4). The opposing relationship between Mg/Ca and Sr/Ca and Ba/Ca along PC2, as well as the negative correlation between Mg/Ca and these elements (see also Fig. S6) suggests that variations in Mg/Ca primarily reflect changes in dissolution of dolomite or other Mg-rich phases from the host rock rather than PCP. White Moon Cave is developed in the San Vicente Creek marble deposit which is part of the metamorphosed Sur Series and is locally interbedded and bounded by schist. These rocks were intruded by quartz diorite, and are overlain by a series of Miocene sandstones and shales. Within the carbonate, some Mg is locally present in silicates and dolomite, especially at the northern end of the San Vicente deposit near the cave^30^. On average, marbles of the Sur Series contain ~ 3.5 wt. % MgO, but this can be as high as 9 wt. %^31^. Slower weathering of calcite than dolomite has been documented experimentally^32^ and in karst systems in the field^33^. Drip water will tend to reach supersaturation with respect to calcite before dolomite^34^, and slower flow rates can increase the amount of dolomite dissolved and thus the amount of Mg in solution^35, 36^. This may be accompanied by a decrease in Sr in solution as dolomite typically contains less Sr than calcite^35, 36^.

This interpretation is consistent with the behavior of Mg/Ca, Sr/Ca, and Ba/Ca, as variations in the proportion of dolomite versus calcite dissolution due to changes in water residence time should lead to such opposing trends in speleothem Mg/Ca with Sr/Ca and Ba/Ca^35^. Variable dissolution of limestone and dolomite marble has also been interpreted as an important control on speleothem Mg/Ca in caves within the Sierra Nevada foothills in California where speleothem Mg/Ca and Sr/Ca show negative correlations^24, 37^, similar to what is noted in WMC1. Furthermore, the elongated columnar crystal fabric observed throughout WMC1 is common in speleothems precipitating from seepage waters that have interacted with dolomite or other Mg-rich rocks^11, 12^. The overall lower Mg/Ca during the core of the 8.2 ka event (Fig. 3) suggests decreased dolomite dissolution, consistent with a wetter climate. PC1 is controlled by variations in P/Ca that co-occur with changes in Zn/Ca and Y/Ca. These elements are associated with soil-derived organics in drip water, likely from decaying plants in the soil zone above the cave^38^. Additionally, high fluxes of metals including Zn and Y in drip waters have been associated with short-lived pulses of infiltration that also transport particulate organic matter^39^. Thus, we interpret the sharp increases in P/Ca present in the WMC1 record during the 8.2 ka event as reflecting periods of rapid infiltration from the soil zone above WMC. Periods of increased P/Ca coeval with negative shifts in δ^13^C and decreased Mg/Ca therefore point to intervals of increased soil inputs and decreased host rock dissolution and prior calcite precipitation, consistent with periods of increased water supply at the height of the 8.2 ka event (Fig. 3)^35, 38, 40^.

## Discussion and Conclusions

Our new record of the climatic response to the 8.2 ka event along the west coast of North America near 37° N latitude shows shifts in several climate proxies that suggest this event was characterized by high but variable infiltration, suggesting an overall wet climate punctuated by larger infiltration events. Shifts in the WMC1 proxy records occur that are closely contemporaneous with other records of the 8.2 ka event. For example, a negative shift in WMC1 δ^13^C that precedes the 8.2 ka event, near 8320 cal yr BP, is coeval within uncertainties of a negative *δ* ^18^O excursion in the speleothem record from Kaite Cave in Spain that has been interpreted to record the first pulse of meltwater from proglacial Lake Agassiz between 8350 and 8340 cal yr BP^41^ (Fig. 2). Excursions in *δ* ^18^O values in speleothems from monsoon regions of the northern and southern hemispheres reveal an abrupt weakening of the Asian monsoon and strengthening of the South American monsoon at ca. 8250–8200 cal yrs BP, when WMC1 Mg/Ca indicates sustained wetter conditions (Fig. 3). Within the core 8.2 ka event, decreases in WMC1 Mg/Ca are matched by increases in P/Ca. The two-step decrease in Mg/Ca in the WMC1 record within the 8.2 ka event period bears similarities to the variability noted in Greenland ice core records^2^ and the high resolution Heshang Cave *δ* ^18^O record of ref. 42 (Fig. 3), suggesting similar timing of responses of East Asian monsoon strength and precipitation on the west coast of North America during this climatic event.

We interpret the WMC1 record to indicate that the 8.2 ka event was expressed on the California coast by increased effective moisture. The high amplitude variability in the WMC δ^13^C record is most prominent between ~8190 and 8110, synchronous with the central period of Asian monsoon weakening and drying noted in the Heshang Cave record. The coupled WMC1 δ^13^C and P/Ca records indicate episodic intervals of rapid infiltration, consistent with more frequent or intense storms on the central California coast during the core of the 8.2 ka event. However, the changes during the 8.2 ka event in some WMC proxies, such as Mg/Ca, are small in amplitude compared to the overall variability in the WMC1 records and that in other global records of the event (Fig. 2). This suggests that, although the influence of the 8.2 ka event was felt on the west coast of North America, the event was not as pronounced there as it was in the high northern latitudes or monsoon-influenced areas. Furthermore, although the WMC1 record may reflect intervals of increased North Pacific sourced vapor to the region, our record is consistent with recent findings that precipitation *δ* ^18^O in this region is subject to complex controls that are challenging to disentangle in coastal areas where the amplitude of *δ* ^18^O changes is small^27^.

Transient climate model simulations suggest that the intensity of the winter storm track over the eastern Pacific was sensitive to changes in meltwater flux to the North Atlantic during the last deglaciation^43^. Meltwater pulses can lead to a more intense and wetter storm track through alteration of the meridional temperature gradient over the Pacific. Although most models do not show a significant change in precipitation in western North America in response to hosing experiments^43, 44^, others have suggested precipitation increases along the central California coast in response to freshwater hosing^45^. Our results are consistent with increased precipitation at the height of the 8.2 ka event, which was likely triggered by draining of lakes Agassiz and Ojibway^3^. Thus, we suggest that this freshwater pulse led to an intensification of the eastern Pacific winter storm track that resulted in periods of intensified rainfall on the central California coast. However, the influence of this intensified storm track on rainfall further inland remains to be documented.

Comparison of the WMC1 record with records of Greenland temperature and Asian monsoon strength suggest that near-synchronous changes in atmospheric circulation occurred across the Pacific in response to these freshwater inputs and the resulting cooling in the North Atlantic region. Significant correlations between speleothem records of Asian monsoon variability, Greenland ice core records, and speleothem *δ* ^18^O records from western North America suggest strong teleconnections between these regions during the last deglaciation^21^. High latitude cooling, possibly associated with increased sea ice extent^46^, could influence monsoon systems in both hemispheres and precipitation in western North America via a southward shift in the ITCZ and strengthening of the northern Hadley cell and the winter northern subtropical jet^47^. The WMC1 record of the 8.2 ka event indicates that the relationship between high latitude cooling, decreased Asian monsoon strength, and increased precipitation in western North America persisted into the early Holocene.

## Methods

### U-series Chronology

Eleven subsamples for ^230^Th/U dating were collected from WMC1. U-series samples were dissolved in 7 N HNO_3_ and equilibrated with a mixed spike containing ^229^Th, ^233^U, and ^236^U. Separation of U and Th was completed with a two-stage HNO_3_-HCl cation exchange procedure, followed by treatment with a mixture of HNO_3_ and HClO_4_ to remove any residual organic material. U and Th fractions were analyzed on a Thermo Neptune Plus Multi-collector ICP-MS. Measured peak heights were corrected for peak tailing, multiplier dark noise/Faraday baselines, instrumental backgrounds, ion counter yields, mass fractionation, interfering spike isotopes, and procedural blanks. Mass fractionation was determined using the gravimetrically determined ^233^U/^236^U ratio of the spike. Activity ratios and ages were calculated using the half-lives of ref. 48 for ^238^U, ref. 49 for ^232^Th, and ref. 50 for ^230^Th and ^234^U.

Three samples, AC-U2, AC-U3, and AC-U4, have high ^232^Th concentrations and unfavorable ^232^Th/^238^U ratios (^232^Th/^238^U > 0.001); thus, they are deemed unsuitable for precise, accurate U-Th dating. Nonetheless, they can be used to estimate the appropriate detritus correction^51^. To examine the effect of detritus composition on calculated ages, corrections for detrital U and Th were made assuming detritus with activity ratios of either (^232^Th/^238^U) = 1.21 ± 0.6, (^230^Th/^238^U) = 1.0 ± 0.1, and (^234^U/^238^U) = 1.0 ± 0.1; or (^232^Th/^238^U) = 0.67 ± 0.34, (^230^Th/^238^U) = 1.0 ± 0.1, and (^234^U/^238^U) = 1.0 ± 0.1 (see Table S1). Applying the latter values brings the calculated ages of the three detritus-rich samples (AC-U2, AC-U3, and AC-U4) into agreement within uncertainties with the age model defined by the relatively pure calcite samples (see Figure S4). This suggests that a detritus correction with (^232^Th/^238^U) = 0.67 ± 0.34 is appropriate for WMC1 and we therefore adopt it as our preferred value for all samples. We note that the dates of the pure samples are not sensitive to the choice of detritus correction, and vary only within their uncertainties regardless of which detritus correction is applied (Table S1). The age-depth model was generated using the StalAge algorithm^15^.

### Speleothem proxy record construction

Samples for stable isotope analysis (*δ* ^18^O and *δ* ^13^C) were milled along the growth axis from one face of the quartered stalagmite using a CM-2 micromill or a handheld dental drill, at ~1 mm spatial resolution for the early Holocene portion of the stalagmite. Further sampling at 200 μm spatial resolution, yielding sub- to multi-annual temporal resolution, was conducted across the portion of the stalagmite that grew during the 8.2 ka event. Stable isotope samples were packed in weigh paper envelopes, and sent to the Stable Isotope Biogeochemistry Lab at Stanford University. There, the samples were analyzed using a Thermo Finnigan Deltaplus XL coupled to a GasBench. Typical precision of stable isotope measurements is <0.2‰ for both oxygen and carbon. Final δ^13^C and δ^18^O values are expressed relative to the international standard V-PDB (Vienna PeeDee Belemnite).

Trace element concentrations were analyzed on thick sections across this same portion of the stalagmite by laser ablation ICP-MS a Photon Machines Excimer laser coupled to a Thermo Finnigan iCapQ at Vanderbilt University. Analyses were conducted as either line scans or lines of individual spots. The lines of spots, conducted through the 8.2 ka event interval, from ~8050–8255 calendar years BP, were done using 25 × 150 μm rectangular slit at 20 μm spacing using 15% laser power and a repetition rate of 10 Hz. The line scans were conducted using a rectangular 20 × 100 μm rectangular slit at a scan speed of 5 μm/s at 20% laser power and a repetition rate of 15 Hz. The line scans followed a pre-ablations step that was conducted over the sample path at a speed of 10 μm/s at 50% laser power and a repetition rate of 15 Hz. The multi-element synthetic glass standard, NIST SRM 612 and the MACS3 synthetic pressed aragonite powder were analyzed at the beginning and end of each run. The NIST SRM 612 glass standard was used for elemental quantification. The data was processed using the Iolite software package. Gaps in the WMC1 trace element record result from mm-scale portions of the growth axis that were lost during cutting of billets for thin sections. Principal components analysis was conducted on log-transformed trace element data using the FactoMineR package in R^52^.

### Water analysis

Drip water samples were collected at three locations within White Moon Cave in March and December of 2015 and March of 2016. Water samples for O and H isotope analysis were collected in acid-cleaned 20 ml LDPE vials and capped with minimal headspace to reduce the potential for evaporative bias. Water samples for carbon isotope analysis of DIC were filtered through 0.2 micron sterile filters and injected in the field into He flushed Labco vials containing phosphoric acid. Water samples were kept refrigerated until analysis.

Integrated monthly precipitation samples were collected at the Long Marine Laboratory in Santa Cruz, CA following the methods of ref. 53. One-liter Nalgene containers were pre-filled with a 1-cm thick layer of mineral oil to eliminate the potential for evaporation after precipitation events and covered with a metal mesh filter to minimize debris entering the sample container. Each month, collectors were sealed, replaced and transported upright to the Santa Clara University Stable Isotope Laboratory for processing. Water samples were extracted from beneath the oil layer with a syringe and passed through multiple paper filters to eliminate oil contamination of the water sample. The stable isotope composition of drip and meteoric water samples was determined using off-axis integrated cavity output spectroscopy with a Los Gatos Research TWIA-45EP water isotope analyzer. Each measurement consisted of five preparatory injections to minimize memory effects and five measured injections. Samples were measured in at least triplicate and corrected using internal and external (USGS) reference water standards. δ^18^O and δ^2^H values are reported relative to Vienna Standard Mean Ocean Water (VSMOW). Replicate analyses demonstrated the typical precision of this technique to be <0.2‰ for δ^18^O and <1‰ for δ^2^H (1σ).

Water samples were analyzed for δ^13^C at the UC Davis Stable Isotope Facility using a Thermo Finnigan Delta V Plus IRMS. Evolved CO_2_ was purged from the Labco vials through a double-needle sampler into a helium carrier stream (20 mL/min). The gas was sampled using a six-port rotary valve (Valco, Houston TX) with either a 100 µL, 50 µL, or 10 µL loop programmed to switch at the maximum CO_2_ concentration in the helium carrier. The CO_2_ was passed to the IRMS through a Poroplot Q GC column (25 m × 0.32 mm ID, 45 °C, 2.5 mL/min). A reference CO_2_ peak was used to calculate provisional delta values of the sample CO_2_ peak. Final δ^13^C values are obtained after adjusting the provisional values for changes in linearity and instrumental drift such that correct δ^13^C values for laboratory reference materials are obtained. At least two laboratory reference materials were analyzed with every 10 samples. Laboratory reference materials are lithium carbonate dissolved in degassed deionized water and a deep seawater (both calibrated against NIST 8545). Final δ^13^C values are expressed relative to the international standard V-PDB.

### Data Availability Statement

Data from this study will be archived with the NOAA National Centers for Environmental Information (www.ncdc.noaa.gov).

## Electronic supplementary material

Supplementary Material
