# Supplementary material for: Climate response to the 8.2 ka event in coastal California
Source: Sci Rep. 2017 Jun 20;7:3886. doi: 10.1038/s41598-017-04215-5 (PMC5478618; doi:10.1038/s41598-017-04215-5)
Supplement: Supplementary file 1 — Supplementary Material [file 41598_2017_4215_MOESM1_ESM.pdf]

This file contains supplementary material for:

Climate response to the 8.2 ka event in coastal California

Jessica L. Oster[1], Warren D. Sharp[2], Aaron K. Covey[1], Jansen Gibson[1], Bruce Rogers[3], and Hari Mix[4]
[1]Department of Earth and Environmental Sciences, Vanderbilt University, 2301 Vanderbilt Place, PMB 351805, Nashville, TN 37235
[2]Berkeley Geochronology Center, 2455 Ridge Road, Berkeley, CA 94709
[3]Western Cave Conservancy
[4]Department of Environmental Studies and Sciences, Santa Clara University, Santa Clara, CA 95053

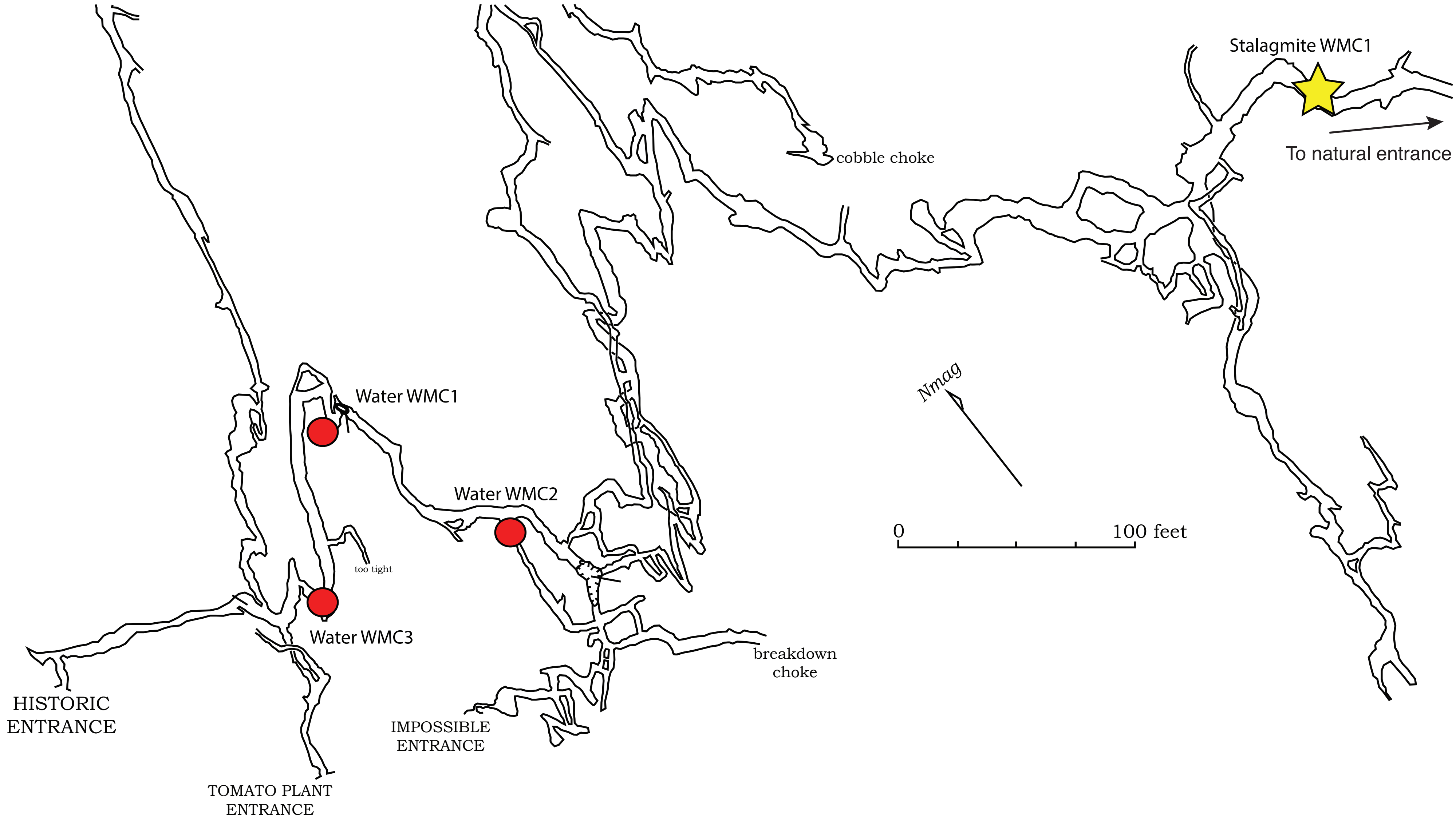

Figure S1: Partial, simplified map of White Moon Cave near Santa Cruz, CA. Red dots show location of drip water samples. Yellow star shows growth location of stalagmite WMC1. The Historic Entrance is located in the wall of a quarry. A natural entrance is located >120m from the stalagmite sample location, off of this simplified map. Map created in Adobe Illustrator by B. Rogers (co-author) based on cartography by P. Bosted and B. Rogers completed between 1986 and 2016 (unpublished).

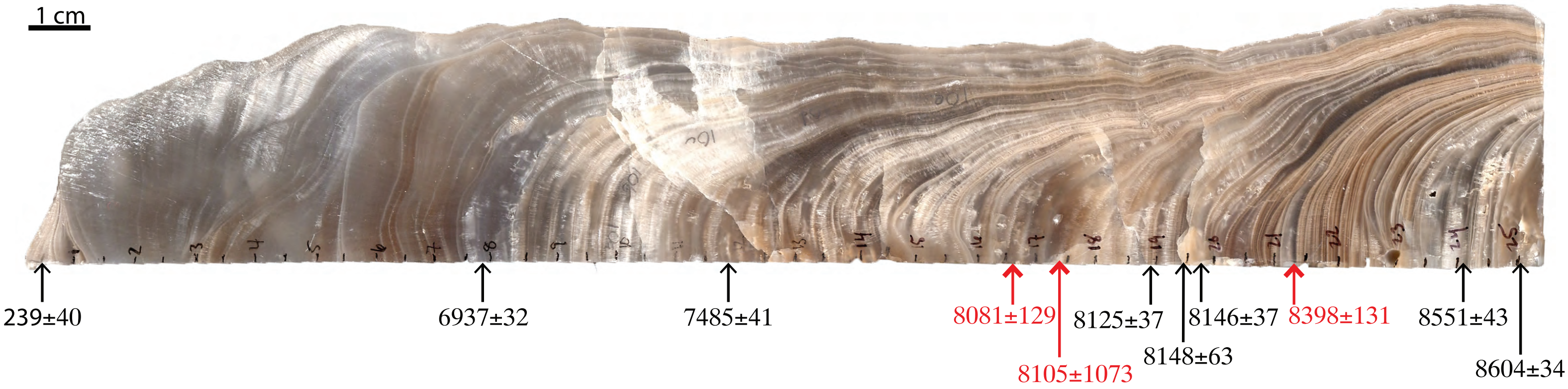

Fig. S2: Image of quartered and polished stalagmite WMC1 with U-series dates (cal BP 1950) and associated 2 sigma errors. Dates shown in red are from samples with high ^{232}Th. As a result, these dates are model-dependant, have high uncertainties, and were not used in the age model.

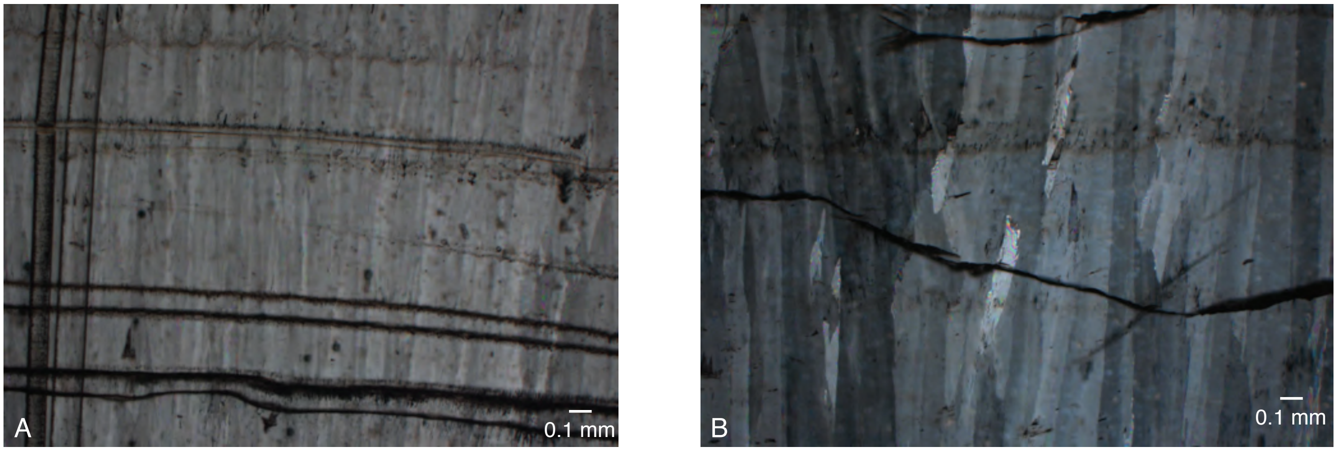

Figure S3: Cross polarized light images of stalagmite WMC1 at 5X magnification. Growth direction is upwards in both images. A) is near 23 cm depth from the top, preceding the 8.2 ka event period. The lines cross-cutting the banding are from laser ablation line scans and are 100 and 125 μm across. Dark lines perpendicular to growth appear to consist of silicate detritus. B) is taken within the 8.2 event period. Irregular lines cross-cutting the images are fractures that occured during thin-sectioning. This fabric is interpreted as elongated columnar fabric (Frisia, et al., *J. Sediment. Res.* 70, 1183 (2000); Frisia, *Int. J. Speleology* 44, 1 (2015).

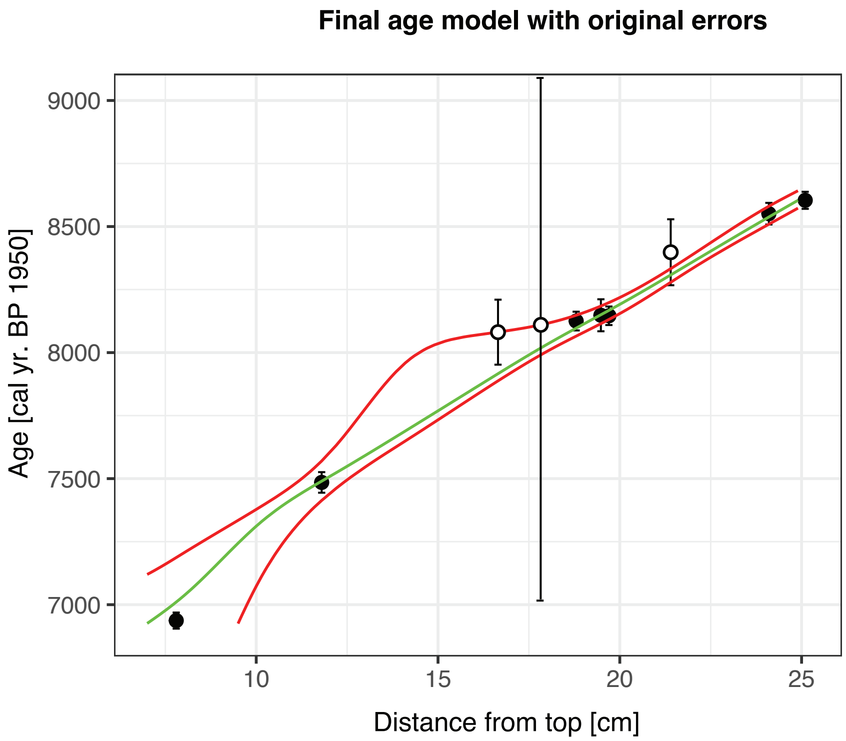

Fig. S4: Age model for studied interval of WMC1 constructed with StalAge (Scholz and Hoffman, 2011) along with 95% confidence limits (red lines). White circles show high ^{232}Th samples not used in constructing the final age model. The youngest age, SC-1, is not shown.

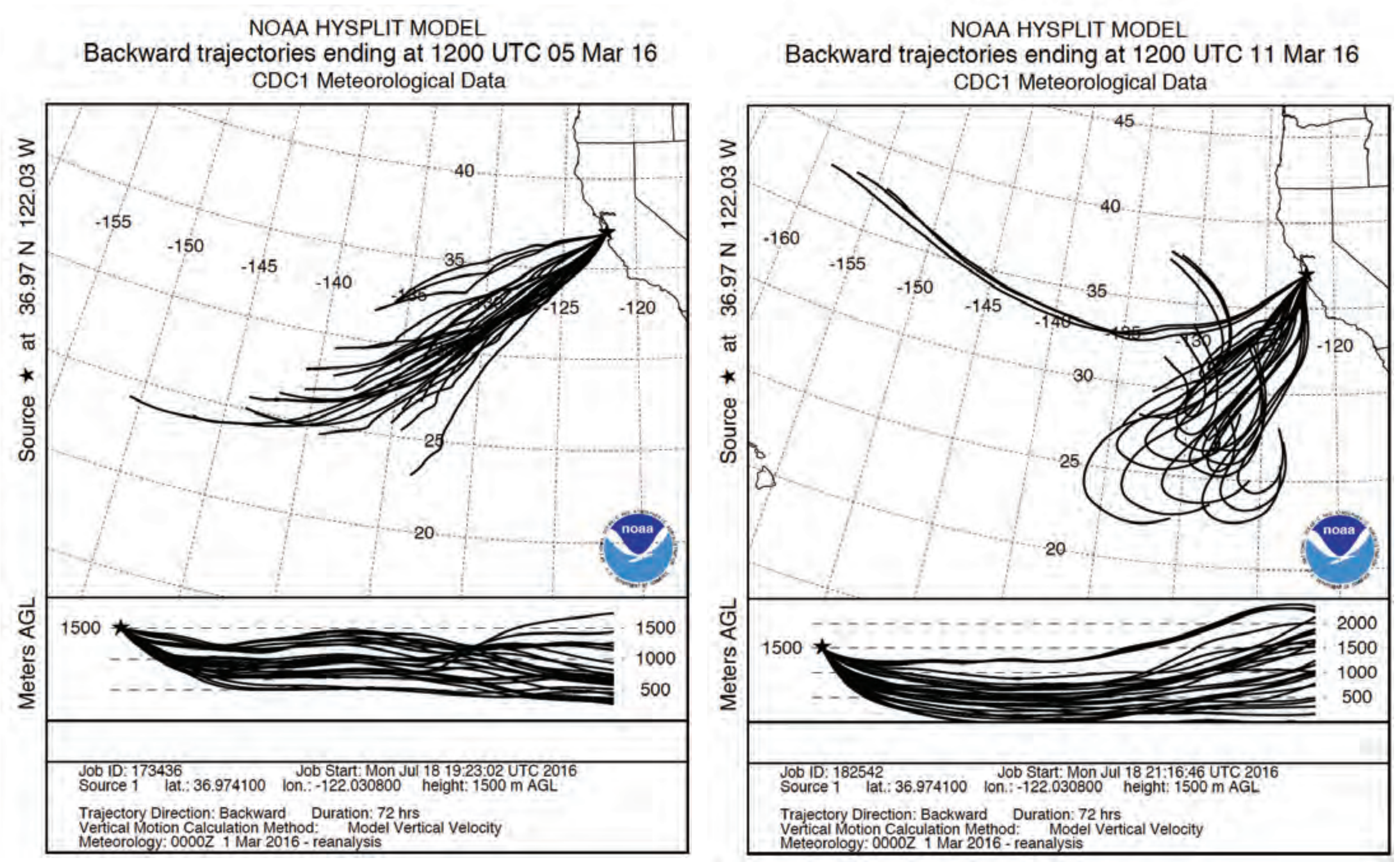

Fig. S5: 72 hour back trajectory ensembles for the two largest rain days in March 2016. Trajectories were initiated at 1500 m a.s.l. Despite the origination of these trajectories to the southwest of WMC, which suggests a subtropical origin for moisture associated with these rain events, the measured oxygen isotope value of precipitation for March 2016 was the most negative monthly average analyzed between December 2015 and March 2016 (see supplementary tables). Trajectories were generated using HYSPLIT (Stein, A.F., Draxler, R.R, Rolph, G.D., Stunder, B.J.B., Cohen, M.D., and Ngan, F., (2015). NOAA's HYSPLIT atmospheric transport and dispersion modeling system, Bull. Amer. Meteor. Soc., 96, 2059-2077, http://dx.doi.org/10.1175/BAMS-D-14-00110.1).

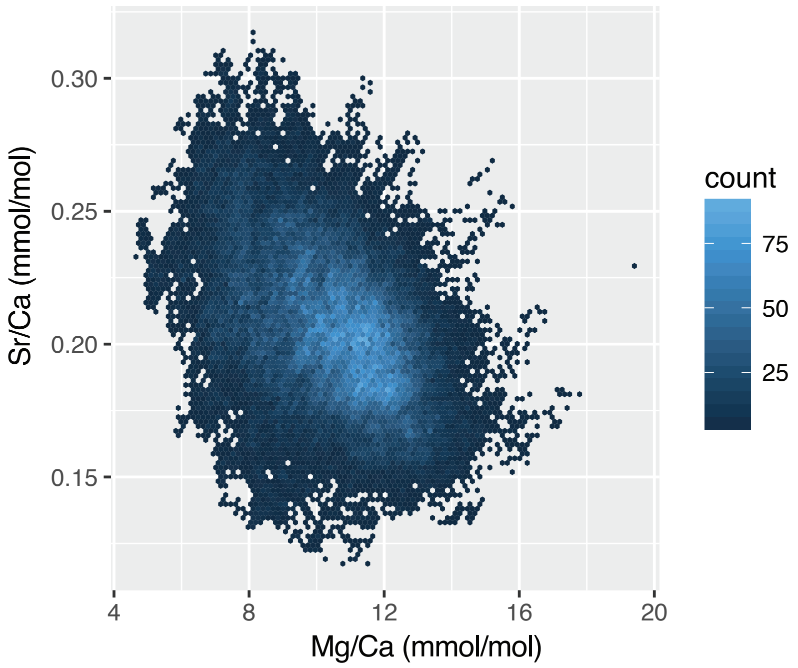

Figure S6: Mg/Ca versus Sr/Ca for the WMC1 stalagmite. Shading shows number of points in each area of the plot. Blue shades indicate increased numbers.

Table S1. U-series analytical data and ages for stalagmite WMC1.

Sample Name	Depth (cm)	Sample Wt. (mg)	U (ppb)	^{232}Th (ppb)	(^{230}Th /^{232}Th)	(^{232}Th /^{238}U)	± (%)	(^{230}Th /^{238}U)	± (%)	(^{234}U /^{238}U)	± (%)	Uncorrected Age, Error (ka)	Detritus-Corrected Age, Error (ka) (^{232}Th/^{238}U)$_{Detr.}$ = 1.21	Detritus-Corrected Age, Error (ka) (^{232}Th/^{238}U)$_{Detr.}$ = 0.67	Detritus-Corrected Age, Error (cal yr BP) (^{232}Th/^{238}U)$_{Detr.}$ = 0.67	Initial (^{234}U /^{238}U)
SC-1	0.10	20.2	1423	3.382	7.251	0.00048	0.13	0.003491	1.93	1.0080	0.28	0.611 ±0.012	0.335 ±0.023	0.301 ±0.040	239 ±40	1.0080 ±0.0028
SC-2	7.8	20.1	3230	2.374	269.0	0.00024	0.13	0.06346	0.26	1.0154	0.23	7.145 ±0.027	7.016 ±0.028	6.999 ±0.032	6937 ±32	1.0157 ±0.0024
SC-3	11.8	19.9	4123	3.632	239.3	0.00028	0.19	0.06801	0.36	1.0110	0.22	7.680 ±0.036	7.568 ±0.036	7.547 ±0.041	7485 ±41	1.0113 ±0.0023
AC-U3*	16.65	33.22	4156	16.49	58.04	0.00129	0.63	0.07503	0.74	1.0100	0.42	8.353 ±0.077	8.239 ±0.094	8.15 ±0.13	8081 ±129	1.0102 ±0.0044
AC-U2*	17.45	50.05	4092	165.4	7.094	0.01298	0.52	0.09209	0.48	1.0224	0.39	10.246 ±0.069	9.10 ±0.59	8.2 ±1.1	8105 ±1073	1.0232 ±0.0047
AC-U5	18.8	58.87	6095	2.882	480.8	0.00015	0.23	0.07395	0.35	1.0167	0.22	8.215 ±0.037	8.201 ±0.037	8.190 ±0.037	8125 ±37	1.0171 ±0.0022
AC-U1	19.48	45.43	6640	1.151	1331	0.00006	0.52	0.07441	0.52	1.0209	0.47	8.222 ±0.063	8.217 ±0.063	8.213 ±0.063	8148 ±63	1.0214 ±0.0049
SC-6	19.7	20.82	5846	4.486	297	0.00025	0.09	0.07405	0.34	1.016	0.11	8.249 ±0.032	8.227 ±0.033	8.209 ±0.037	8146 ±37	1.0167 ±0.0012
AC-U4*	21.4	43.21	6292	26.22	57.76	0.00135	0.48	0.07802	0.58	1.0160	0.52	8.679 ±0.075	8.560 ±0.093	8.46 ±0.13	8398 ±131	1.0164 ±0.0055
SC-5	24.1	21.39	9661	11.82	195.8	0.00040	0.15	0.07781	0.29	1.0170	0.11	8.678 ±0.029	8.643 ±0.033	8.614 ±0.043	8551 ±43	1.0174 ±0.0012
SC-4	25.1	19.6	7145	1.153	1487	0.00005	0.12	0.07770	0.33	1.0159	0.18	8.725 ±0.035	8.670 ±0.034	8.666 ±0.034	8604 ±34	1.0163 ±0.0018

All isotope ratios are activity ratios. Uncertainties are given at 2 standard deviations. (*) denotes samples with ^{232}Th/^{238}U ratios >0.001, whose ages are deemed unreliable. Uncorrected ages are calculated from measured ratios. Ages were corrected for U and Th from detritus using (^{232}Th/^{238}U) = 1.21 ± 0.60 or 0.67 ± 0.34, (^{230}Th/^{238}U) = 1.0 ± 0.1, and (^{234}U/^{238}U) = 1.0 ± 0.1. Decay constants are those of Jaffey (1971) for ^{238}U and Cheng et al. (2013) for ^{230}Th and ^{234}U. Initial (^{234}U/^{238}U) is back-calculated from the measured ratio and the corrected ^{230}Th age using (^{232}Th/^{238}U) = 0.67 ± 0.34. Age (cal yr BP) is age before 1950.

Table S2: Measured drip water stable isotope values at White Moon Cave

Drip Site	Date of Collection	$\delta^{2}H_{VSMOW}$ (‰)	$\delta^{18}O_{VSMOW}$ (‰)	$\delta^{13}C_{VPDB}$ (‰)
WMC1	3/4/15	-33.1	-5.6	
WMC1	12/16/15	-31.11	-5.41	-3.48
WMC1	3/6/16	-28.54	-4.76	-5.21
WMC2	3/4/15	-30.7	-5.2	
WMC2	12/16/15			-2.73
WMC2	3/6/16	-28.47	-4.87	-7.15
WMC3	12/16/15	-34.27	-5.85	-5.95
WMC3	3/6/16	-28.02	-5.01	-7.23

Table S3: Monthly average rainwater stable isotope data for 2015-2016 at Santa Cruz, CA

Month	dD	dD stdev	d18O	d18O stdev	n	Avg Temp (degC)	Precip Amt. (cm)
December	-10.97	0.92	-3.51	0.14	3	10.49	16.33
January	-29.82	0.42	-4.01	0.17	3	12.25	29.97
February	-14.36	1.15	-3.03	0.22	4	12.57	2.61
March	-31.48	0.53	-5.37	0.06	4	13.29	19.43
